# Use of venoarterial extracorporeal membrane oxygenation in fulminant chagasic myocarditis as a bridge to heart transplant

**DOI:** 10.5935/0103-507X.20150066

**Published:** 2015

**Authors:** André Rodrigues Durães, Fernando Augusto Marinho dos Santos Figueira, André Rabelo Lafayette, Juliana de Castro Solano Martins, Sá Juliano Cavalcante de

**Affiliations:** 1 Universidade do Estado da Bahia - Salvador (BA), Brazil.; 2 Instituto de Medicina Integral Professor Fernando Figueira - Recife (PE), Brazil.

**Keywords:** Extracorporeal membrane oxygenation, Chagas cardiomyopathy, Heart transplantation, Case reports

## Abstract

A 17-year-old Brazilian male presented with progressive dyspnea for 15 days, worsening in the last 24 hours, and was admitted in respiratory failure and cardiogenic shock, with multiple organ dysfunctions. Echocardiography showed a left ventricle ejection fraction of 11%, severe diffuse hypokinesia, and a systolic pulmonary artery pressure of 50mmHg, resulting in the need for hemodynamic support with dobutamine (20mcg/kg/min) and noradrenaline (1.7mcg/kg/min). After 48 hours with no clinical or hemodynamic improvement, an extracorporeal membrane oxygenation was implanted. The patient presented with hemodynamic, systemic perfusion and renal and liver function improvements; however, his cardiac function did not recover after 72 hours, and he was transfer to another hospital. Air transport was conducted from Salvador to Recife in Brazil. A heart transplant was performed with rapid recovery of both liver and kidney functions, as well as good graft function. Histopathology of the explanted heart showed chronic active myocarditis and amastigotes of *Trypanosoma cruzi*. The estimated global prevalence of *T. cruzi* infections declined from 18 million in 1991, when the first regional control initiative began, to 5.7 million in 2010. Myocarditis is an inflammatory disease due to infectious or non-infectious conditions. Clinical manifestation is variable, ranging from subclinical presentation to refractory heart failure and cardiogenic shock. Several reports suggest that the use of extracorporeal membrane oxygenation in patients presenting with severe refractory myocarditis is a potential bridging therapy to heart transplant when there is no spontaneous recovery of ventricular function. In a 6-month follow-up outpatient consult, the patient presented well and was asymptomatic.

## Abstract (pt)

Jovem com 17 anos de idade atendido com dispneia progressiva há 15 dias e piora nas últimas 24 horas. Foi admitido em estado de insuficiência respiratória e choque cardiogênico com disfunção de múltiplos órgãos. O ecocardiograma mostrou fração de ejeção ventricular esquerda de 11%, grave hipocinesia difusa e pressão sistólica da artéria pulmonar de 50mmHg. Houve necessidade de suporte hemodinâmico com uso de dobutamina (20mcg/kg/minuto) e noradrenalina (1,7mcg/kg/minuto). Após 48 horas, o paciente não apresentou melhora hemodinâmica nem clínica, optando-se, então, pela implantação de membrana de oxigenação extracorpórea. Ocorreu melhora do ponto de vista hemodinâmico, da perfusão sistêmica, da função renal e hepática, porém, após 72 horas, não houve recuperação da função cardíaca. Optou-se, assim, pela transferência para outro hospital. O paciente foi transferido por transporte aéreo de Salvador (BA) para Recife (PE). Foi realizado transplante cardíaco com rápida recuperação da função hepática e renal, e boa função do enxerto. A histopatologia do coração explantado demonstrou miocardite crônica ativa e amastigotas de *Trypanosoma cruzi*. A prevalência global estimada de infecção por *T. cruzi* caiu de 18 milhões em 1991, quando a primeira iniciativa regional de controle teve início, para 5,7 milhões em 2010. A miocardite é uma doença inflamatória causada por condições infecciosas ou não infecciosas. As manifestações clínicas variam desde um quadro subclínico até insuficiência cardíaca e choque cardiogênico. Diversos relatos sugerem que o uso de membrana de oxigenação extracorpórea em pacientes com quadro grave e refratário de miocardite é uma opção como terapia ponte até transplante cardíaco, nos casos sem recuperação espontânea da função ventricular. Em uma consulta ambulatorial de acompanhamento realizada 6 meses após o transplante, o paciente encontrava-se bem e assintomático.

## INTRODUCTION

Extracorporeal membrane oxygenation (ECMO) for acute heart failure in adults can be used as a bridge to myocardial recovery, cardiac transplantation or implantation of a left ventricular assist device.^(1)^

There are two main types of ECMO: venovenous and venoarterial; venoarterial is the method of choice for acute heart failure in adults.^(1)^ The ECMO system, a modified heart-lung machine, generally consists of a centrifugal pump, a heat exchanger and a membrane oxygenator. Desaturated venous blood is aspirated from the right atrium into a centrifugal pump through a long steel wire-reinforced cannula inserted into the right atrium via the femoral vein. The pump outflow is directed into a membrane oxygenator, and it is guided via an outflow cannula into the descending aorta via the femoral artery.^(2)^ Typical ECMO complications include systemic inflammatory response syndrome, renal failure, limb ischemia and bleeding.^(2)^

## CASE REPORT

A 17-year-old Brazilian male presented with progressive dyspnea for 15 days, worsening in the last 24 hours, and was admitted in respiratory failure and cardiogenic shock, with multiple organ dysfunctions, to the cardiac intensive care unit at the *Hospital Ana Nery*, Salvador-Brazil. Echocardiography showed a left ventricle ejection fraction of 11%, severe diffuse hypokinesia and a systolic pulmonary artery pressure of 50mmHg, which resulted in the need for hemodynamic support with dobutamine (20mcg/kg/min) and noradrenaline (1.7mcg/kg/min).

After 36 hours with no clinical or hemodynamic improvement, peripheral veno-arterial extracorporeal membrane oxygenation (VA-ECMO) was implanted using a centrifuge magnetic pump with a polymethylpentene oxygenation membrane (Rotaflow Centrifugal Pump^®^/Quadrox-i Adult/Bioline coated/MAQUET Cardiopulmonary AG, Hirrlingen, Germany). Heparin treatment (100UI/kg bolus and 20UI/Kg/h) was initiated. Blood flow was initially at 4.571mL/min, with 6.000mL/min gas flow (pure oxygen Sweeper). Hemodynamic (noradrenaline dropped to 0.21mcg/kg/min), systemic perfusion (lactate of 17mmol/L dropped to 2.5mmol/L and increase diuresis volume), and renal (serum creatinine from 3.5 to 1.2mg/dL) and liver (international normalized ratio 7.4 to 3.1) function improvement were evident in 24 hours (Table 1). After 36 hours, ventilator-associated pneumonia was suspected (progressive infiltrate on chest radiograph, leukocytosis and purulent tracheobronchial secretions), and teicoplanin and meropenem were initiated. Within 72 hours of ECMO use, no cardiac function improvement was noted and a transfer to the *Instituto de Medicina Integral Professor Fernando Figueira* (IMIP) Transplant Center in Recife, Brazil was then executed.

**Table 1:** Clinical and hemodynamic parameters before and after extracorporeal membrane oxygenation implantation*

	Before	After
Noradrenalin (mcg/kg/min)	1.7	0.2
Dobutamine (mcg/kg/min)	20	20
Mean blood pressure (mmHg)	45	66
Heart rate (bpm)	148	110
Lactate (mmol/L)	17	2.5
Bicarbonate	14	21
pH	7.28	7.34
Diuresis (mL/Kg/h)	0	1.4
Serum creatinine (mg/dL)	3.5	2.1
INR	7.43	3.21
AST	7599	3077
ALT	6242	4217
Serum potassium (mg/dL)	7.6	4.5
Ventilator FiO_2_ (%)	30	21
pO_2_	114.8	253
PCO2	35.3	38
SVO_2_ (%)	65	72

INR - international normalized ratio; AST - aspartate aminotransferase; ALT - alanine aminotransferase; FiO_2_ - inspired oxygen fraction; SVO_2_ - mixed venous oxygen saturation.

* Interval of 24 hours before and after implantation.

**Figure 1:**
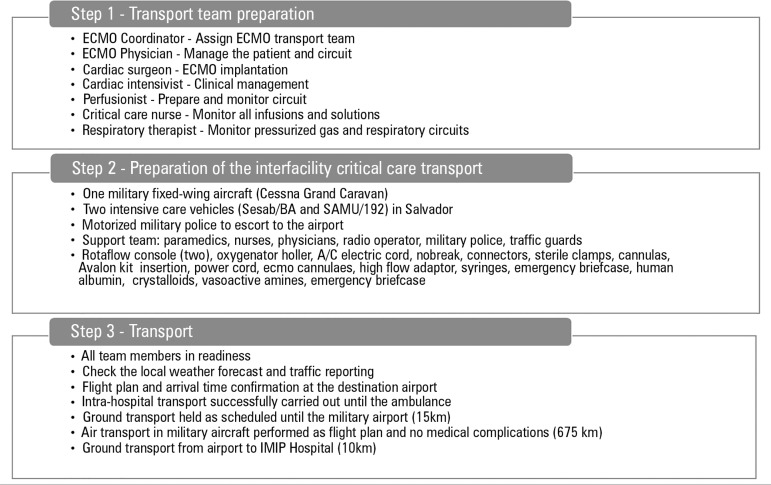
Logistics of inter-hospital transport between Salvador and Recife (Brazil). ECMO - extracorporeal membrane oxygenation; SESAB - *Secretaria Estadual de Saúde da Bahia*; SAMU - *Serviço Móvel de Urgência*; IMIP - *Instituto de Medicina Integral Professor Fernando Figueira*.

**Figure 2:**
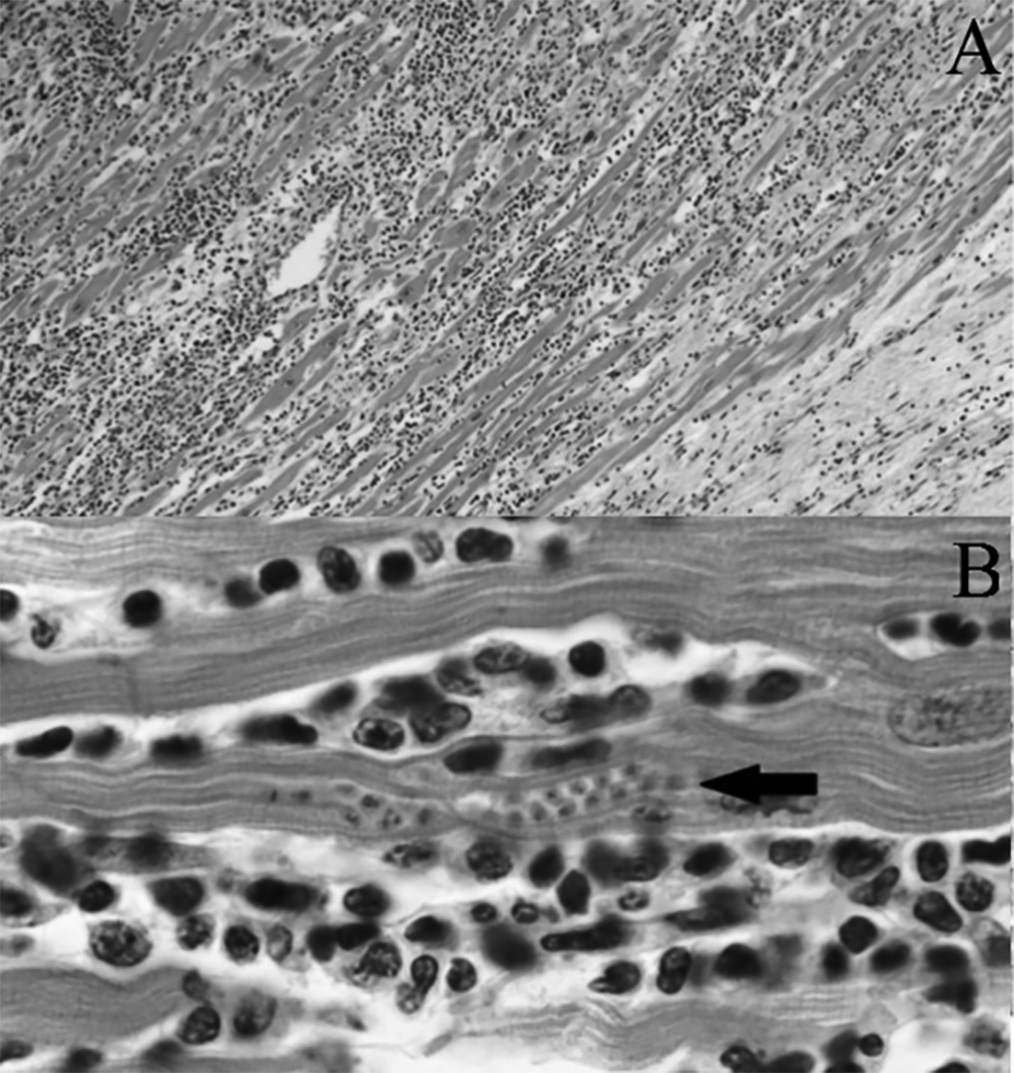
Histology of an explanted heart sample. A) Photomicrograph (x400) of an hematoxylin and eosin stained sample. B) At this magnification (x1000), the organisms (amastigotes of *Trypanosoma cruzi*) within a myocyte (arrow) and the adjacent inflammatory response are more clearly observed.

Air transport using a military plane was provided from Salvador to Recife 4 days after admission (travelled distance of 675km) (Figure 1S in electronic supplementary material). The logistics of inter-hospital transport involved approximately 50 professionals from different specialties, including physicians, nurses, physiotherapists, paramedics, perfusionists and police officers due to the lack of a specialized team trained in ECMO transport (Figure 1). Heart transplant with intraoperative ECMO decannulation was performed after 48 hours, with excellent performance, rapid recovery of the liver and kidney functions, good graft function and successful weaning off circulatory support. Histopathology of the explanted heart showed chronic active myocarditis and amastigotes of *Trypanosoma cruzi* (Figure 2), presenting chagasic cardiomyopathy.

## DISCUSSION

The estimated global prevalence of *T. cruzi* infections declined from 18 million in 1991, when the first regional control initiative began, to 5.7 million in 2010.^(3)^ Myocarditis is an inflammatory disease due to infectious or non-infectious conditions. Clinical manifestation is variable, ranging from subclinical presentations to refractory heart failure and cardiogenic shock.^(4)^ Mechanical circulatory support with an intra-aortic balloon or ventricular assist devices should be considered in cases refractory to medical therapy.^(4)^ Several reports suggest that the use of ECMO in patients presenting with severe refractory myocarditis is a potential bridging therapy to heart transplantation when there is no spontaneous recovery of ventricular function.^(5,6)^ The VA-ECMO can be percutaneously implanted at the bedside and can be kept for several weeks with proper care. It is the preferred device when patients present with biventricular dysfunction and often promotes rapid improvement of the hemodynamic status, oxygenation parameters and organ function.^(7,8)^ In the face of heart function improvement, the mechanical support device can be progressively withdrawn, but when cardiac function impairment is maintained, the treatment of choice would be a long-term device or heart transplant.^(9,10)^ In a 6-month follow-up outpatient consult, the patient presented himself in good health, ratifying the cost-utility of this procedure in Brazil.^(10)^

## CONCLUSION

In the presented case, veno-arterial extracorporeal membrane oxygenation was used on a patient with multiple organ failure secondary to a defined and refractory cardiogenic shock. The benefit of the method was unquestionable, even in the advanced stages of renal and liver failure. The patient presented with a partial recovery of the affected systems, making possible the implementation of the definitive therapy to severe myocarditis or heart transplant. Long- and short-distance inter-hospital transport on extracorporeal membrane oxygenation can be safely performed, but it demands a subspecialized team, highly competent in intensive care and be aware of the risks involved in transporting these patients.
