# Supplementary material for: Use of venoarterial extracorporeal membrane oxygenation in fulminant chagasic myocarditis as a bridge to heart transplant
Source: Rev Bras Ter Intensiva. 2015 Oct-Dec;27(4):397–401. doi: 10.5935/0103-507X.20150066 (PMC4738827; doi:10.5935/0103-507X.20150066)
Supplement: Supplementary file 1 [file rbti-27-04-0397-suppl01.pdf]

Use of venoarterial extracorporeal membrane oxygenation in fulminant chagasic myocarditis as a bridge to heart transplant

Uso de membrana de oxigenação extracorpórea venoarterial em um caso de miocardite chagásica fulminante como ponte para transplante cardíaco

André Rodrigues Durães[1], Fernando Augusto Marinho dos Santos Figueira[2], André Rabelo Lafayette[2], Juliana de Castro Solano Martins[1], Juliano Cavalcante de Sá[1]

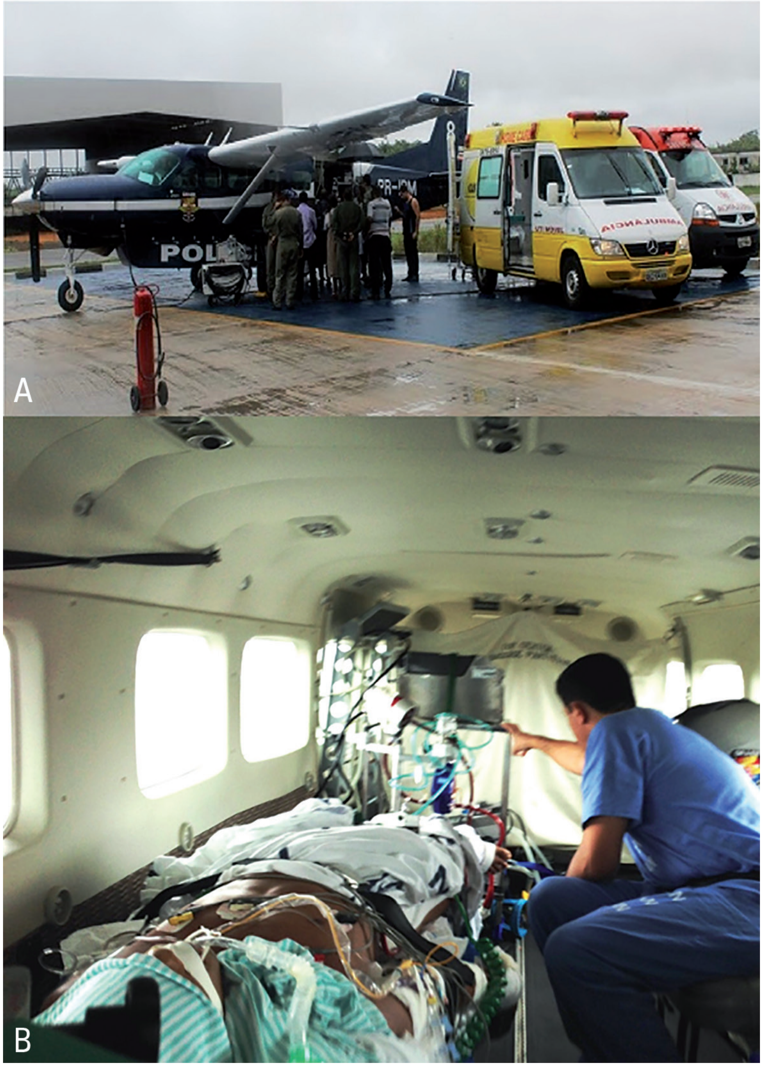

Figure 1S - A) Patient transport between the ground ambulance and aircraft. B) Patient and extracorporeal membrane oxygenation equipment positioning during air transport from Salvador to Recife (Brazil).
